# Transactions of the Chicago Society of Physicians and Surgeons, January 26th, 1874

**Published:** 1874-02-15

**Authors:** Plym. S. Hayes


### TRANSACTIONS OF THE CHICAGO SOCIETY OF PHYSICIANS AND SURGEONS.

### Meeting of January 26th, 1874.

*Reported by Plym. S. Hayes, M.D.*

The Society met as usual, in the parlor of the Grand Pacific Hotel, the President, Dr. Fisher, in the chair.

Drs. E. Andrews and W. Blanchard were elected to membership.

The Secretary read a paper by the President, Dr. Fisher, on the progress of medicine. The paper reviewed the advances made in medical science to the present time, and compared the slow progress of this science before the commencement of the nineteenth century, with its rapid advance since that time.

Dr. C. P. Simon reported the case of a young man who had taken about three drachms of tinct. opium. When discovered, three hours before the doctor was summoned, the body was motionless and pulseless. When the doctor arrived, he found the extremities cyanosed and cold; the neck and lower jaw rigid; the pupils dilated; and the iris brilliant and phosphorescent. No pulse could be detected; and there were no respiiations. Upon applying the ear to the chest, the doctor thought he was able to detect pulsations, which averaged thirty to the minute. Ten minutes after his arrival the pulsations had entirely ceased.

Dr. Etheridge then related the following case of opium poisoning. When he first saw the patient, there were twelve stertorous respirations, and forty-eight pulsations, to the minute. After a time the respirations ceased entirely for about eighty seconds, after which they were resumed with an audible sound; and then the respirations, which were quiet and normal, gradually grew more and more shallow and stertorous, until they again ceased. The periods of cessation varied from fifty to one hundred seconds; while those of respiration continued to take place from eight to ten minutes. During the cessation, there was a tremor of the intercostal muscles. The restorative means used were the stomach-pump, and atropia given by the mouth. Half an hour after the atropia had been given, the pupils graually dilated until death occurred, eight hours after the doctor had been called.

Dr. Trimble cited two similar cases in which atropia and artificial respiration had been used; in one of the cases faradization had also been employed; both recovered.

Dr. Wilder related the case of a man who took of tinct. opium two drachms. Although emetics, belladonna, artificial respiration and friction were used, the patient died. The pupils dilated after the belldonna had been given.

Dr. Bartlett mentioned a case of poisoning from a belladonna plaster, which had been applied for threatened mammary abscess. The patient was taking morphine all of the time. He also related the case of a child who had taken one drachm tinct. opium. He was called soon after the drug had been taken, and not having a stomach-pump, filled the stomach with water, and then reversed the child, when the contents of the stomach escaped.

Dr. Etheridge stated that larger doses of opium and belladonna could be borne when given together, than when given separately, the toxic effect of the one neutralizing that of the other, and cited Dr. Brown-Sequard as authority.

Dr. Powell remarked that he uses, after an operation, one-half grain of morphia, and one-sixtieth grain of atropia, hypodermically; the action of the morphia being thus continued much longer than when given alone.

Dr. Trimble introduced a resolution requiring the President to appoint a committee of three on necrology at each annual meeting.

Dr. Danforth is expected to read, at the next meeting, a paper on the pathology of the late cholera endemic, illustrated by means of a solar microscope.

The Society then adjourned.

---

A DIFFICULTY IN FŒTAL AUSCULTATION.—Dr. J. Braxton Hicks calls attention to a point with regard to the diagnosis of pregnancy and the life of the fœtus, by means of the existence of the fœtal heart-sounds—which he had not unfrequently observed in the course of his practice, but which he does not remember to have seen in print—and summed up his observations as follows: First, that the number of vibrations of the abdominal muscles in a state of half-suspension can be distinctly counted, watch in hand; second, that their number and sound is so like those of a very rapid fœtal heart that they may be mistaken for them.—*Philadelphia Medical Reporter*.

